# Depth Image–Based Deep Learning of Grasp Planning for Textureless Planar-Faced Objects in Vision-Guided Robotic Bin-Picking

**DOI:** 10.3390/s20030706

**Published:** 2020-01-28

**Authors:** Ping Jiang, Yoshiyuki Ishihara, Nobukatsu Sugiyama, Junji Oaki, Seiji Tokura, Atsushi Sugahara, Akihito Ogawa

**Affiliations:** Corporate Research & Development Center, Toshiba Corporation, 1, Komukai-Toshiba-cho, Saiwai-ku, Kawasaki 212-8582, Japan

**Keywords:** deep learning, bin picking, grasp planning, textureless, visual servoing

## Abstract

Bin-picking of small parcels and other textureless planar-faced objects is a common task at warehouses. A general color image–based vision-guided robot picking system requires feature extraction and goal image preparation of various objects. However, feature extraction for goal image matching is difficult for textureless objects. Further, prior preparation of huge numbers of goal images is impractical at a warehouse. In this paper, we propose a novel depth image–based vision-guided robot bin-picking system for textureless planar-faced objects. Our method uses a deep convolutional neural network (DCNN) model that is trained on 15,000 annotated depth images synthetically generated in a physics simulator to directly predict grasp points without object segmentation. Unlike previous studies that predicted grasp points for a robot suction hand with only one vacuum cup, our DCNN also predicts optimal grasp patterns for a hand with two vacuum cups (left cup on, right cup on, or both cups on). Further, we propose a surface feature descriptor to extract surface features (center position and normal) and refine the predicted grasp point position, removing the need for texture features for vision-guided robot control and sim-to-real modification for DCNN model training. Experimental results demonstrate the efficiency of our system, namely that a robot with 7 degrees of freedom can pick randomly posed textureless boxes in a cluttered environment with a 97.5% success rate at speeds exceeding 1000 pieces per hour.

## 1. Introduction

Demand for logistics workers in Japan is high and still rising, making automatic robot manipulation systems crucially important for overcoming labor shortages [1]. Picking textureless planar-faced objects is a common robot manipulation task for item transfer in warehouses.

Vision-guided robot picking tasks entail grasp planning to determine a target object and detect its grasp points, which are then used to track and pick the target object by visual servoing (VS) [2], an image-based visual-guided robot control scheme. Examples of VS include position-based VS [3,4] and hybrid VS [5,6,7]), which is flexible due to its robustness to camera calibration errors. VS generally requires a textured goal image for feature matching to estimate the relative pose of an object with respect to the camera [8], but many items in a warehouse, such as cardboard boxes, are textureless. Even for textured items, it is impractical and time-consuming to prepare textured goal images of various items in a warehouse. Bin picking in a warehouse is even more challenging, because items in a bin are often in disorganized heaps, making it difficult to infer their relative poses in textured color images due to sensor noise, obstructions, and occlusions [9]. Therefore, using depth images, thereby eliminating the need for goal images and extraction of image texture features, is preferred for picking planar-faced objects in cluttered environments.

In combination with VS, grasp planning determines the grasp order and grasp points of a target object. The graspability scores of grasp points are ranked, and an optimal target is selected as the target point for the grasp attempt. Grasp planning methods can be broadly divided into one- and two-stage methods. Two-stage methods first conduct object segmentation and pose estimation, then search for grasp points on the target object. Object segmentation can be conducted by traditional template matching based on designed descriptors (e.g., scale-invariant feature transform (SIFT) [10,11]) or deep convolutional neural networks (e.g., mask region-based CNN (R-CNN) [12], Single shot multibox detector (SSD) [13], You Only Look Once (YOLO) [14], or SegNet [15] for 2D object segmentation, and PointNet [16] or PointCNN [17] for 3D object segmentation). Given the segmented points, object pose can be estimated by cloud point registration, which searches a spatial transform to align two cloud sets [18]. Drost et al. [19] proposed Point Pair Features (PPF) for building a hash table as object model global descriptors and recovering 6D poses from a depth image by a voting scheme, the performance of which under noise and occlusion was improved in References [20,21], and Reference [18]. Recently, deep convolutional neural networks that jointly output the semantic segmentation and object 6D pose have been proposed [18,22,23,24]. These methods integrate object segmentation and pose estimation, and outperform conventional methods in cluttered scenes. Once the object pose is obtained and the object is known, a template-based method can be used to find corresponding grasp points predefined in a database, but such databases are not always affordable in a warehouse. As an alternative, analytical methods can analyze kinematics and dynamics of grasp candidates to rank the best grasp points [25], but doing so requires foreknowledge of certain criteria, such as object geometry, physics models, and force analytics [26,27], which might be unavailable or difficult to design for bin picking in cluttered scenes.

Unlike two-stage methods, a one-stage method [28,29,30,31,32,33,34,35,36,37], namely, one-shot grasp detection, directly regresses grasp points and their classes without object segmentation or pose estimation. This method is preferable for object picking in a warehouse for two reasons. First, in most cases of random bin picking, the goal is to grasp and place as many objects as possible per hour rather than to grasp a specific object in a pile, so segmentation is unnecessary. Second, one-stage methods are faster than two-stage methods. One-shot detection methods are thus best suited to real-time grasp detection [27]. Levine et al. [29] were among the first to incorporate a convolutional neural network (CNN) model that directly predicts the probability of success of a given motor command in visually guided grasp planning. Douglas et al. [36] proposed Generative Grasping Convolutional Neural Networks (GG-CNN) to predict the quality and pose of grasps at every pixel with fast prediction speeds of 19 ms. Zeng et al. [37] directly predicted the affordance map of four primitive grasp actions based on RGB-D images. However, these studies required large datasets. Johns et al. [5] showed that the major challenge with deep learning is the need for very large amounts of training data, and thus they opted to generate and use simulated data for the training process [38].

Faced with this challenge, recent works [30,31,32,33,34,35] have trained CNN models by data generated in simulations. Josh et al. [30] used domain randomization and generative models to predict scores for grasp candidates, but only tested their method on single-object grasping. Bousmalis et al. [31] proposed GraspGAN, a pixel-level domain adaptation model, to predict the grasp probability of a motion command input for bin picking, but this model’s success rate is still relatively low. Mahler et al. [32,33] proposed Grasp-Quality CNN (GQ-CNN) to predict grasp robustness and to rank most grasp points. They generated grasp points in a simulator and evaluated the robustness of each using a metric named Robust Ferrari–Canny. Alexandre et al. [34] extended this method to a Grasp Quality Spatial Transformer Network (GQ-STN), which improves the precision of grasp robustness predictions. Ulrich et al. [35] proposed a CNN model that uses simulated depth images to predict the remaining cost-to-go of moving to the nearest viable grasp, but execution times were so long that each grasp attempt took 20–30 s.

Although the above studies have proposed CNN models that are trained by simulated images to predict grasp points, the predictions are affected by differences between simulated and actual images. Domain randomization is effective but cannot totally eliminate the influences of unrealistic datasets. Further, these studies used a two-finger gripper or a one-vacuum-cup suction hand. To the best of our knowledge, no previous studies have used CNN models to predict grasp patterns (vacuum cup activation selection) for a two-vacuum-cup suction hand.

In this paper, we propose a novel depth-image-based vision-guided robot bin-picking system for textureless planar-faced objects for robots with a two-vacuum-cup suction hand. We propose a deep convolutional neural network (DCNN) trained on 15,000 annotated depth images synthetically generated in a physics simulator [39] to predict grasp points (centers of graspable surfaces) and their grasp patterns, namely, the suction hand vacuum-cup activation mode: left cup on (L), right cup on (R), or both cups on (B). Additionally, we incorporate a surface feature descriptor that extracts surface features (center position and normal) of the surface containing the predicted grasp point, eliminating the need for VS texture features and goal images. Because the surface feature descriptor reconstructs the surface during feature extraction, the predicted grasp point position can be refined, which helps to reduce the influence of unrealistic datasets without using a sim-to-real method.

Our primary contributions are as follows:

- We propose a framework for automatically generating a training dataset in consideration of the grasp pattern for planar-faced object picking in Gazebo [39], which is more efficient and convenient than manual collection.
- We propose a DCNN model to simultaneously predict grasp point positions and their corresponding grasp patterns (vacuum-cup activation modes) for a two-vacuum-cup suction hand.
- We incorporate surface-feature descriptors for DCNN prediction refinement and feature extraction, allowing the system to be free of sim-to-real refinement for DCNN predictions, as well as texture features and goal images for VS.

## 2. Picking Robot

**Figure 1:**
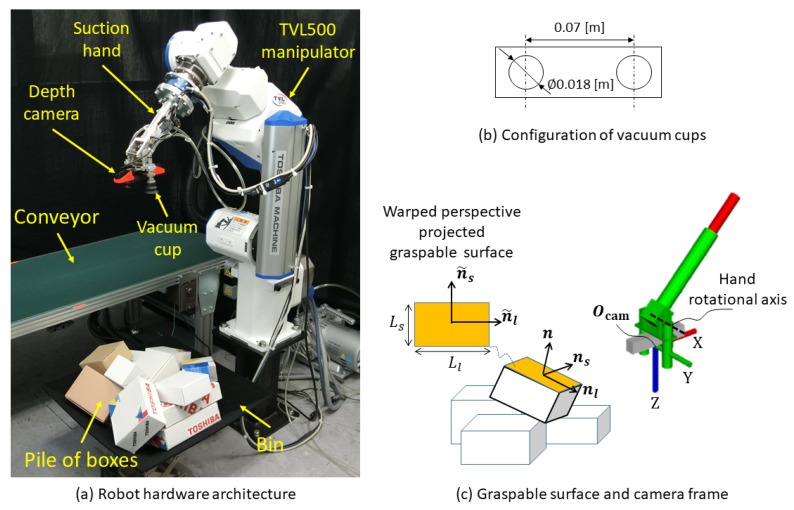
Picking robot. (**a**) Robot hardware architecture. (**b**) Configuration of vacuum cups. (**c**) Graspable surface and camera frame. n, $n_{l}$, and $n_{s}$ respectively indicate the normal, major (long) axis, and minor (short) axis of the graspable surface in the camera frame. $L_{l}$ and $L_{s}$ are respectively the long-side and short-side lengths of the graspable surface in the camera frame. ${\tilde{n}}_{l}$ and ${\tilde{n}}_{s}$ are respectively the major-axis and minor-axis directions of a warped perspective projected on the graspable surface.

In this study, we focus on grasping with a two-vacuum-cup suction hand amid a pile of boxes, which are the most common planar-faced objects in a warehouse. As shown in Figure 1a, the picking robot consists of a 6-degree-of-freedom (DoF) manipulator (TVL500; Toshiba Machine Co. Ltd., Shizuoka, Japan) and a 1-DoF suction hand (Figure 1c). The suction hand is an eye-in-hand system having two 0.018-m diameter vacuum cups (Figure 1b), which are symmetrically set across an RGB-D camera (RealSense SR300; Intel, Santa Clara, CA, USA). This camera can capture both RGB color and depth images, but our system uses only the depth images; RGB images are used only to visualize the results.

As shown in Figure 1c, we define the normal, major (long)-axis, and minor (short)-axis directions for the graspable surface of a planar-faced object in the camera frame as n, $n_{l}$, and $n_{s}$, respectively. $L_{l}$ and $L_{s}$ are respectively the long- and short-side lengths of the surface. ${\tilde{n}}_{l}$ and ${\tilde{n}}_{s}$ are respectively the major-axis and minor-axis directions of a warped perspective projected on the graspable surface.

## 3. Vision-Guided Robot Bin-Picking System

**Figure 2:**
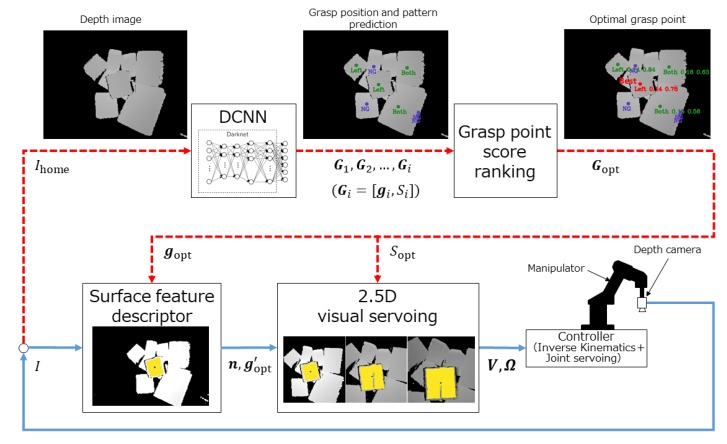
System diagram. The red dashed lines indicate the loop for grasp planning for textureless planar-faced objects by a deep convolutional neural network (DCNN) model at the robot’s home position. The blue solid lines indicate the visual guided robot control loop. Both loops only use depth images. *I* is the depth image, and $I_{\mathrm{home}}$ is the depth image captured at the robot’s home position. $G_{i}$ is the *i*th predicted grasp point, defined as the set of grasp point position g and grasp pattern *S*. $G_{\mathrm{opt}}$ is the optimal grasp point among all predicted points, and $g_{\mathrm{opt}}$ and $S_{\mathrm{opt}}$ indicate its corresponding position and grasp pattern, respectively. n and ${g^{\prime }}_{\mathrm{opt}}$ are respectively the surface normal and refined center position, namely the surface feature. V and Ω are respectively the reference translational and rotational velocity of the suction hand.

As shown in Figure 2, the proposed system comprises a learning-based textureless planar-faced object grasp planning loop (red dashed lines) and a visual guided robot control loop (blue solid lines). Both loops only use depth images as inputs. The grasp planning loop uses a DCNN model trained on a simulated dataset to predict grasp points G (center points of visible surfaces). Unlike previous methods that output grasp point positions only, here G includes a grasp point position g and its corresponding grasp pattern *S* for determination of vacuum-cup activation. The predicted grasp points are further scored according to distance to the camera and the angle between the point normal at g and camera axis *Z*. These scores are ranked and the optimal grasp point $G_{\mathrm{opt}}$ is sent to the robot control loop. Section 4.2 presents details of the grasp point score ranking.

Given position $g_{\mathrm{opt}}$ of $G_{\mathrm{opt}}$, the surface descriptor uses $g_{\mathrm{opt}}$ as an initial seeking point to reconstruct the surface including $g_{\mathrm{opt}}$, whose surface features (normal n and center position ${g^{\prime }}_{\mathrm{opt}}$) will then be calculated. During surface reconstruction, $g_{\mathrm{opt}}$ is refined to ${g^{\prime }}_{\mathrm{opt}}$ so that even if the predicted $g_{\mathrm{opt}}$ is inaccurate, the positional offset error can be compensated for. Based on n, ${g^{\prime }}_{\mathrm{opt}}$, and $S_{\mathrm{opt}}$, 2.5D VS calculates reference translational velocity V and rotational velocity Ω of the suction hand for controlling the robot to track and apply suction to the target surface by the predicted vacuum cup activation mode $S_{\mathrm{opt}}$ for the optimal grasp point $G_{\mathrm{opt}}$. Note that the grasp planning loop only activates when the robot is at its home position, because the descriptor can use the calculated ${g^{\prime }}_{\mathrm{opt}}$ from the previous iteration as an initial seeking point for the subsequent iteration.

## 4. Learning-Based Grasp Planning for Textureless Planar-Faced Objects

The learning-based grasp planning loop for textureless planar-faced objects uses a DCNN model (Section 4.1) to predict G, which includes center points of all visible planar surfaces and their grasp patterns (vacuum cup activation modes) in an input depth image. The predicted points are further ranked and the optimal grasp point is output, as described in Section 4.2. Furthermore, we propose a framework (Section 4.3) for automatically collecting the training dataset for DCNN in a physics simulator.

### 4.1. DCNN Model

Supervised learning is conducted to directly learn grasp points without segmentation. The DCNN model regresses grasp point position g and predicts the probability of four classes (grasp pattern *S*) of g, which includes L, R, B, and non-graspable (NG). Here, we consider g as center points of all visible planar surfaces, because the center point of a surface is assumed to be the most stable position to apply suction. Further, the class probability prediction considers the mechanical and kinematic constraints of the robot (e.g., joint limits and inverse kinematics solutions), manipulation efforts, and collision status with the surroundings. Section 4.3 details how we label the class of each g for depth images in the training dataset.

**Figure 3:**
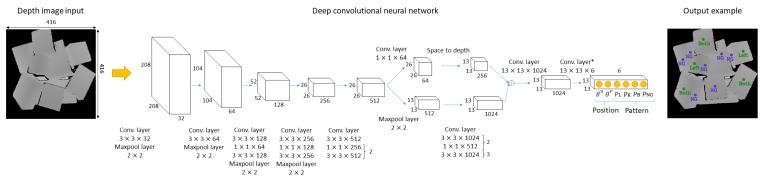
DCNN model.

As shown in Figure 3, the DCNN model has 23 convolutional layers and uses Darknet-23 [40] as the backbone. A leaky rectified linear activation function is used for all convolutional layers except the last ("conv. layer*" in Figure 3). DCNN takes a 416×416 depth image as input and predicts a list of grasping points G where the maximal probability of the four classes exceeds a threshold. Specifically, G is defined as

(1) $$G_{i}=\left[g_{i},S_{i}\right]$$

(2) $$g_{i}=\left[g_{i}^{u},g_{i}^{v}\right],$$

where

(3) $$S_{i}=\max\left(p_{R},p_{L},p_{B},p_{\mathrm{NG}}\right)\;\mathrm{subject}\mathrm{to}\;S_{i}>th,$$

and $G_{i}$ is *i*th grasp point. $g_{i}$ and $S_{i}$ are respectively the position and grasp pattern of $G_{i}$. $g_{i}$ is represented as the pixel coordinate $\left[g_{i}^{u},g_{i}^{v}\right]$ in a depth image with horizontal and vertical axes *u* and *v*. $p_{R},p_{L},p_{B},p_{\mathrm{NG}}$ are probabilities of the R, L, B, and NG classes, respectively. $S_{i}$ is the maximum of these four classes, subject to the constraint that $G_{i}$ will be output only when $S_{i}$ exceeds the DCNN output threshold th.

In this paper, we focus on random picking amid a pile of objects, so focal loss is used because it addresses class imbalance and exhibits high accuracy in dense objects detection [41]. The DCNN model is trained using the following multipart loss functions:

(4) $$L=\lambda_{coord}L_{coord}+\lambda_{class}L_{class}$$

(5) $$L_{coord}=\frac{1}{B}\sum_{j=1}^{N^{2}}1_{j}^{GP}{\left(g_{j}^{u}-{\hat{g}}_{j}^{u}\right)}^{2}+{\left(g_{j}^{v}-{\hat{g}}_{j}^{v}\right)}^{2}$$

(6) $$L_{class}=\frac{1}{B}\sum_{j=1}^{N^{2}}\sum_{c\in classes}\left\{\begin{array}{cc} -\alpha {\left(1-p_{j}\left(c\right)\right)}^{\gamma }log\left(p_{j}\left(c\right)\right) & {\hat{p}}_{j}\left(c\right)=1\\ -\left(1-\alpha \right)p_{j}{\left(c\right)}^{\gamma }log\left(1-p_{j}\left(c\right)\right) & \mathrm{otherwise} \end{array}\right.,$$

with $L_{coord}$ evaluating the positional offset by L2 loss and $L_{class}$ evaluating the multiclass classification by focal loss. *N* (=13) is the grid size of a depth image, which was divided to speed up training. *B* is the number of ground-truth grasp points in the depth image. $g_{j}^{u}$ and $g_{j}^{v}$ are pixel coordinates of the grasp point position in the *j*th cell. *c* is one of the four classes (R, L, B, or NG). $p_{j}\left(c\right)$ is the probability of *c* in the *j*th cell. α and γ are tunable focusing parameters of the focal loss. α and γ are set to 0.25 and 2.0, respectively. $\lambda_{coord}$ and $\lambda_{class}$ are penalty weights for positional offset and classification error, respectively. Both $\lambda_{coord}$ and $\lambda_{class}$ are set to 1.0. ${𝟙}_{j}^{GP}$ is a flag indicating if there is a ground-truth grasp point in the *j*th cell; $L_{coord}$ is evaluated only when ${𝟙}_{j}^{GP}=1$ (a ground-truth grasp point exists).

### 4.2. Grasp Point Score Ranking

This section describes how predicted grasp points G are scored and the optimal point $G_{\mathrm{opt}}$ in G is output. Equation (7) evaluates the graspability score $J_{i}$ for grasp point *i*.

We assume that a higher grasp point, in other words one closer to the camera, is faster and safer to access, so $J_{\mathrm{distance}}$ (Equation (9)) is defined to evaluate the normalized distance from $G_{i}$ to the camera center. We also assume that a smaller surface inclination angle is easier to grasp (because a level surface is more easily grasped than a inclined one), so $J_{\mathrm{inclination}}$ (Equation (10)) is defined to evaluate normalized surface inclination angles. ϕ is the angle between the point normal $n_{G_{i}}$ at $G_{i}$ and the camera-axis *Z* normal $n_{Z}$ ([0,0,1]), as in Equation (8). Further, there is a tradeoff between $J_{\mathrm{distance}}$ and $J_{\mathrm{inclination}}$. We prioritize $J_{\mathrm{distance}}$ if $\phi <\frac{\pi }{6}$ and $J_{\mathrm{inclination}}$ otherwise, because we empirically found that $J_{\mathrm{distance}}$ has a dominant influence on graspability when ϕ is smaller than $\frac{\pi }{6}$, but $J_{\mathrm{inclination}}$ is dominant when ϕ exceeds $\frac{\pi }{6}$.

(7) $$J_{i}=\left\{\begin{array}{cc} J_{\mathrm{distance}} & \phi <\frac{\pi }{6}\\ J_{\mathrm{inclination}} & \mathrm{otherwise} \end{array}\right.$$

(8) $$\phi =\arccos\frac{n_{G_{i}}\cdot n_{Z}}{∥n_{G_{i}}∥∥n_{Z}∥}=\arccos\frac{n_{G_{i}}^{Z}}{∥n_{G_{i}}∥}$$

(9) $$J_{\mathrm{distance}}=1-\frac{I(g_{i}^{u},g_{i}^{v})}{D_{\mathrm{ground}}}$$

(10) $$J_{\mathrm{inclination}}=1-\frac{2\phi }{\pi }.$$

Here, $n_{G_{i}}$ is the point normal at $G_{i}$. $n_{G_{i}}^{Z}$ is the *Z* component of $n_{G_{i}}$, and $n_{Z}$ is the camera axis *Z* normal. $I(g_{i}^{u},g_{i}^{v})$ is the depth at $G_{i}$ in a depth image, which represents the distance between the camera and $G_{i}$ along the camera *Z* axis. $D_{\mathrm{ground}}$ is the distance between the ground and the camera at the robot’s home position.

### 4.3. Automatic Training Dataset Collection

**Figure 4:**
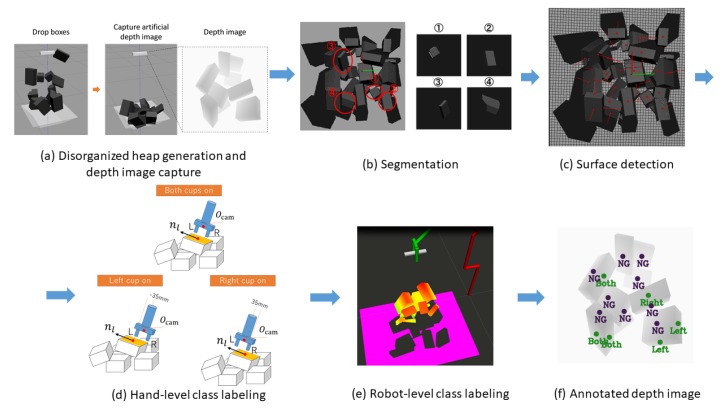
Automatic training dataset collection framework. (**a**) Disorganized heap generation and depth image capture. (**b**) Segmentation. (**c**) Surface detection. (**d**) Hand-level class labeling. (**e**) Robot-level class labeling. (**f**) Annotated depth image.

Figure 4 shows the automatic training dataset collection framework. As shown in Figure 4a, to generate a pile of textureless planar-faced objects with a physically plausible space configuration, we drop a random number (0 to 20) of boxes in random poses from a height of 0.625 m relative to the bin bottom. To generate a disorganized heap we use boxes of three dimensions, determined based on common warehouse items: 0.12×0.075×0.023 m, 0.145×0.092×0.052 m, and 0.06×0.06×0.07 m. Once the boxes reach a stable state, the camera captures an artificial depth image from a height of 0.625 m above the bin center.

To annotate the generated depth image, the center point position of all visible surfaces is required to annotate the grasp point position. Meanwhile, the grasp pattern selection and a graspability check must be performed to label the class for each grasp point. Given a 2.5 D depth image and camera intrinsic matrix, the point cloud can be simply converted from the depth image. Given that the robotic hand is a vacuum type, we assume the grasp point to be the center of a surface for stable suction. To detect the surface, we use a box filter provided by the Point Cloud Library [42] to conduct point cloud segmentation (Figure 4b). Then, for each segmented point cloud cluster, we use the Random Sample Consensus (RANSAC) [43] algorithm to detect the surface and calculate the surface-center position and normal (Figure 4c). Finally, we convert the positions of surface center points from camera coordinates to depth-image pixel coordinates, which are used as the grasp points.

The four classes include three graspable pattern classes (L, R, and B) and one non-graspable pattern class (NG). We first label the classes for each grasp point (Figure 4d) considering only the suction hand, then check whether the planar-faced object can be grasped by the labeled grasp pattern (Figure 4e) considering the entire robot. Such a two-stage class labeling method is used because directly conducting robot-level class labeling is time consuming. Through hand-level class labeling, obviously non-graspable points can be quickly found.

**Figure 5:**
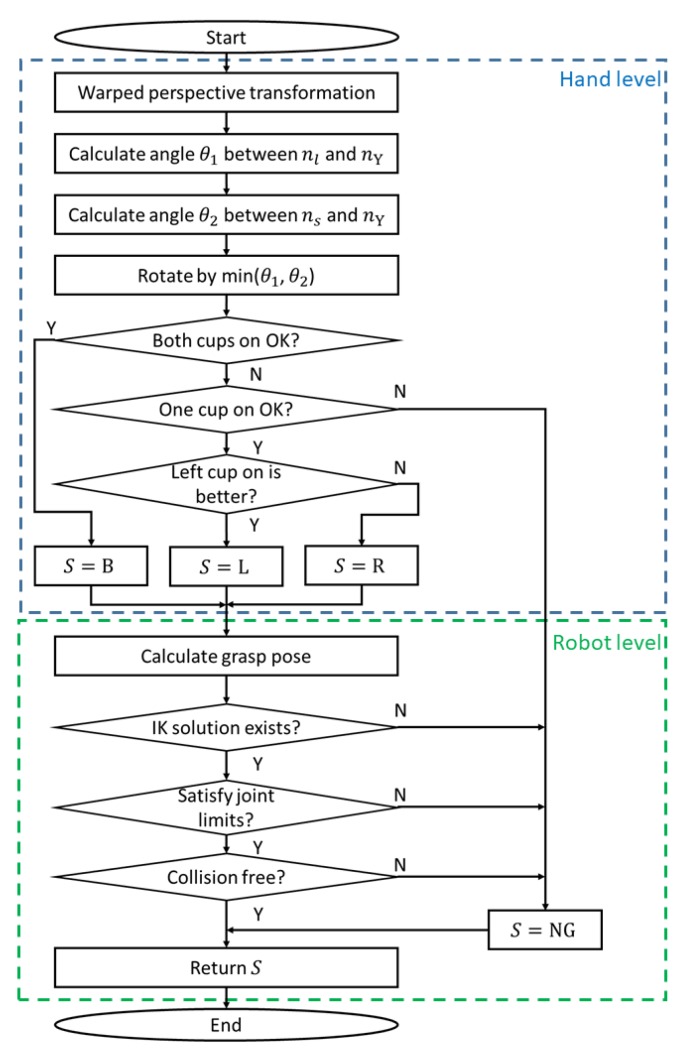
Class-labeling algorithm. The blue dashed line indicates steps for hand-level class labeling. The green dashed line indicates steps for robot-level class labeling. *S* is a grasp pattern, namely, a class label. L, R, B, and non graspable (NG) indicate left cup on, right cup on, both cups on, and non-graspable, respectively. IK indicates an inverse kinematics calculation.

**Figure 6:**
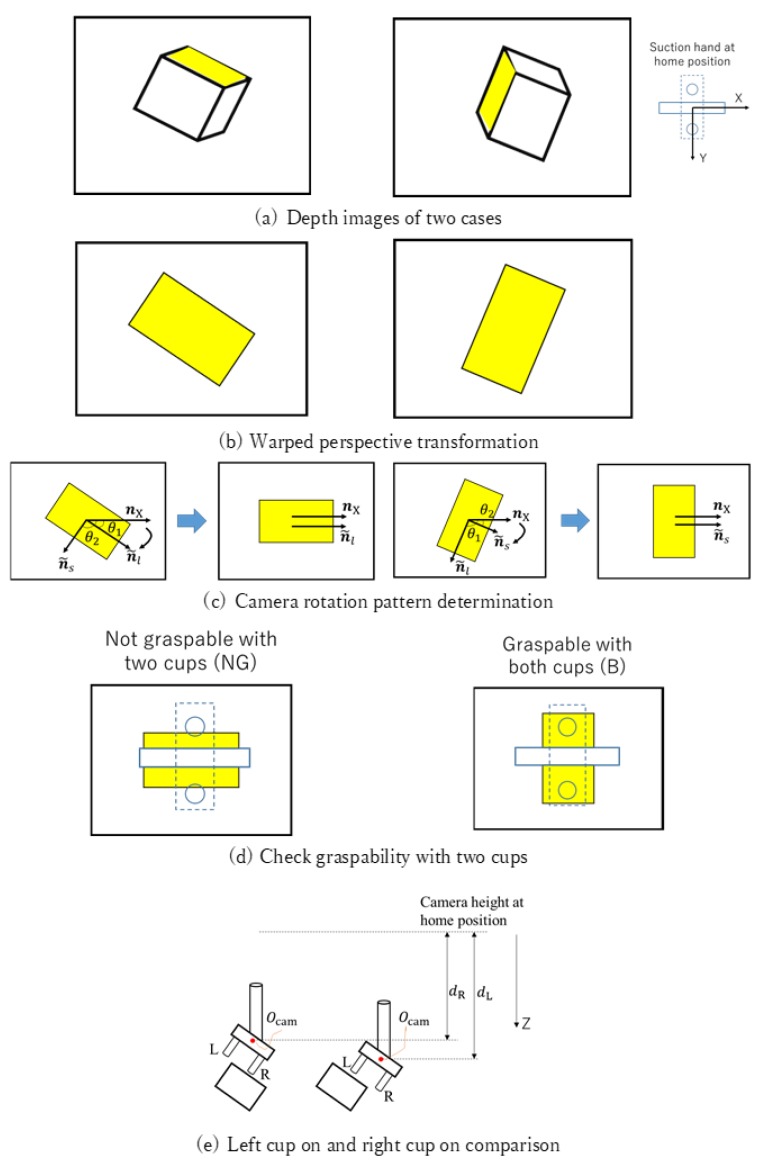
Examples of hand-level class labeling for two depth images. (**a**) Two cases of depth images. *X* is the camera long axis. (**b**) Warped perspective transformation. (**c**) Camera rotation pattern determination. (**d**) Check graspability with two cups. (**e**) Left cup on and right cup on comparison. $O_{cam}$ is the camera center when picking the object. $d_{R}$ and $d_{L}$ are the distance between $O_{cam}$ and the camera center at the home position along camera axis *Z* when picking the object by the corresponding pattern.

Figure 5 shows the class labeling algorithm. Hand-level labeling is first conducted based on the depth image. For ease of understanding, Figure 6 shows examples of the hand-level labeling process. Given a target surface depth image, we perform a warped perspective transformation to let the surface face toward the camera, as shown in Figure 6b. The angle $\theta_{1}$ between the perspective-projected surface long axis ${\tilde{n}}_{l}$ and the camera *X* axis $n_{X}$ together with angle $\theta_{2}$ between the surface short axis ${\tilde{n}}_{s}$ and camera *X* axis $n_{X}$ are calculated. These two angles are calculated to determine the camera rotation pattern shown in Figure 6c. To grasp the object with a smaller manipulation (rotation) effort, the 2D target pose of the camera is aimed at aligning axis *X* with the surface axis having a smaller axis angle with respect to axis *X*. The length of the aligned axis is then calculated to check whether the length is sufficiently long to grasp the object with both vacuum cups (Figure 6d). If not, we further check whether one vacuum cup can reach the object. If one cup can reach the surface, we compare performance when using the left and right cups (Figure 6e). The higher camera center $O_{\mathrm{cam}}$, which is closer to the camera center at the robot home position along axis *Z*, is safer, so the corresponding pattern will be selected as the label. For example, in Figure 6e the left-side $O_{\mathrm{cam}}$ is higher from the bin bottom, so the right-cup pattern will be selected. Hand-level labeling can quickly filter out NG grasp points, which are not needed for further robot-level labeling.

Robot-level labeling checks whether a grasp point is graspable by a target hand pose for the entire robot. During hand-level labeling, we determined the 2D target pose for which camera-axis *X* and *Y* directions had been calculated. To determine the 6D target pose in the camera frame, $O_{\mathrm{cam}}$ and the camera axis *Z* direction are required. The axis *Z* direction can be obtained by calculating the anti-direction of the surface norm. Meanwhile, as Figure 4d shows, $O_{\mathrm{cam}}$ is a 0.15-m offset from the surface center along the camera axis *Z* for both cups in the class, while further offsets of 0.035 or −0.035 m are added along camera axis *Y* for left and right cups, respectively. Note that 0.035 m is the distance from a vacuum cup center to the camera center (see Figure 1b). Given a target suction hand pose, inverse kinematics calculations, a joint-limit check, and a collision check are sequentially conducted to find plausible joint angles for the robot. If no solution exists, the grasp point is labeled as NG; otherwise the hand-level labeling result is kept.

## 5. Visual-Guided Robot Control

As shown in Figure 2, the depth-image-based visual-guided robot control scheme consists of a surface feature descriptor and 2.5D VS. The feature descriptor calculates the center position and normal of the target surface by the region expansion algorithm at each iteration and 2.5D visual servo feedback control is used to calculate the hand velocity for the controller to manipulate the robot to the target pose.

### 5.1. Surface Feature Descriptor

**Figure 7:**
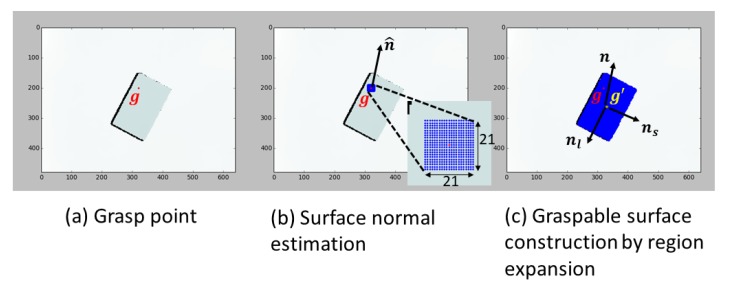
Surface feature descriptor. (**a**) Initially sought grasp point. The red point g is the point $g_{\mathrm{opt}}$ predicted by the learning-based grasp planning loop at the robot home position in the first iteration. From the second iteration, this point will be the calculated surface center $g^{\prime }$ in the previous iteration. (**b**) Surface-normal estimation. $\hat{n}$ is the estimated surface normal. (**c**) Graspable surface reconstruction by region expansion. The blue surface is the constructed graspable surface. The yellow point $g^{\prime }$, namely the refined g, is the surface center.

Figure 7 shows a general framework for the surface feature descriptor. The descriptor performs two functions. The first is to refine $g_{\mathrm{opt}}$ prediction by the learning-based grasp planning loop to $g_{\mathrm{opt}}^{\prime }$, which the surface center $g_{\mathrm{opt}}$ belongs to, at the robot’s home position in the first iteration. The second is to provide the surface center and normal to the 2.5D VS.

To calculate surface features, we use the region-growing algorithm for surface construction, because region growing is more robust than RANSAC in actual applications [44]. To determine the region expansion rule, an initial seeking point g and the normal of the surface to which g belongs must be calculated. g is $g_{\mathrm{opt}}$ at the home position but becomes the calculated $g^{\prime }$ in the previous iteration after the robot starts to move. To determine the normal, we extract a 21×21-pixel window (Figure 7b) centered at g, then fit the plane by calculating the singular value decomposition of cloud points in the window. The plane-fitting problem is then to find all points P satisfying

(11) $$\hat{n}\cdot P\left(u,v\right)+\hat{D}=0$$

in camera coordinates, where $\hat{n}$ is the estimated normal of the fitted plane. P(u,v) is the counterpart of a point (u,v) in a depth image. Namely, P(u,v) is the corresponding point of (u,v) in a point cloud converted from the depth image given the camera-intrinsic matrix.

Then the region expansion rules can be defined to filter out points belonging to the surface (Equation (11)) in the whole depth image as

(12) $$|n_{P}\cdot P\left(u,v\right)+\hat{D}|<\epsilon_{1}$$

(13) $$\left|\right|n_{P}-\hat{n}{\left|\right|}^{2}<\epsilon_{2}$$

(14) $$n_{P}=\left(P\left(u+1,v\right)-P\left(u-1,v\right)\right)\times \left(P\left(u,v+1\right)-P\left(u,v-1\right)\right),$$

where $n_{P}$ is the normal of point P.

Given the fitted plane, we can further simply obtain the surface center (refined grasp point) $g^{\prime }$, surface long axis $n_{l}$, and short axis $n_{s}$. We conducted the warped-perspective transformation to project $n_{l}$ and $n_{s}$ onto ${\tilde{n}}_{l}$ and ${\tilde{n}}_{s}$, which are necessary for VS in Section 5.2.

The surface descriptor finally outputs features including surface normal n in camera coordinates, the warped perspective projected on surface long axis ${\tilde{n}}_{l}$ and short axis ${\tilde{n}}_{s}$ in camera coordinates, and the surface center $g^{\prime }$ in pixel coordinates.

### 5.2. 2.5D Visual Servoing

Velocity control of the gripper in an eye-in-hand system controls the 6 degrees of freedom of the camera. Inspired by 2.5D VS [8], gripper velocity control can be considered as controlling the following feedback error e to be zero:

(15) $$e=\left[\begin{array}{c} p_{e}-p_{e}^{*}\\ u\theta \end{array}\right],$$

where $p_{e}$ is the normalized $g^{\prime }$ from the surface feature descriptor and $p_{e}^{*}$ is the normalized target position. uθ gives the angle/axis parametrization for camera rotation.

$p_{e}$ ([x, y, z]) is a perspective projection of $g^{\prime }$ ([X, Y, Z]) to the graspable surface, as in Equation (16). $p_{e}^{*}$ is the normalized center of the depth image for class B. This is because all points in the depth image captured at the target pose belong to the graspable surface, and thus the image center point is the target, namely the surface center. For classes L and R, respective offsets of −0.035 and 0.035 m are added as follows:

(16) $$p_{e}={\left[x,y,z\right]}^{T}={\left[\frac{X}{Z},\frac{Y}{Z},\log\left(Z\right)\right]}^{T}$$

(17) $$p_{e}^{*}={\left[0,\frac{off}{d^{*}},\log\left(d^{*}\right)\right]}^{T}$$

(18) $$off=\left\{\begin{array}{cc} -0.035 & S=L\\ 0 & S=B\\ 0.035 & S=R \end{array}\right.,$$

where $d^{*}$ is the distance between the camera frame and the graspable surface at the target pose. Specifically, $d^{*}$ indicates the offset distance from $g^{\prime }$ along the n direction.

We use $\dot{e}=-\lambda e$ to make $p_{e}-p_{e}^{*}$ and uθ in Equation (15) converge to zero. As reported in [5,6], $\dot{e}$ can be represented as

(19) $$\dot{e}=\left[\begin{array}{c} {\dot{p}}_{e}\\ u\dot{\theta } \end{array}\right]=\left[\begin{array}{cc} \frac{1}{d^{*}\rho }L_{v} & L_{w}\\ 0 & I_{3} \end{array}\right]\left[\begin{array}{c} V\\ \Omega \end{array}\right]$$

(20) $$L_{v}=\left[\begin{array}{ccc} -1 & 0 & x\\ 0 & -1 & y\\ 0 & 0 & -1 \end{array}\right]$$

(21) $$L_{w}=\left[\begin{array}{ccc} xy & -(1+x^{2}) & y\\ \left(1+y^{2}\right) & -xy & -x\\ -y & x & 0 \end{array}\right]$$

(22) $$\rho =\frac{Z}{d^{*}},$$

where V and Ω are respectively the translational and rotational velocities of the camera. The gripper velocity control law $\dot{e}=-\lambda e$ can thus be described as

(23) $$\left[\begin{array}{c} V\\ \Omega \end{array}\right]=-\lambda {\left[\begin{array}{cc} \frac{1}{d^{*}\rho }L_{v} & L_{w}\\ 0 & I_{3} \end{array}\right]}^{-1}\left[\begin{array}{c} p_{e}-p_{e}^{*}\\ u\theta \end{array}\right],$$

where λ is the convergence rate, which is set to 0.4.

To let camera axis *Z* coincide with graspable surface normal n and camera axis *X* coincide with axis (${\tilde{n}}_{l}$ or ${\tilde{n}}_{s}$) with smaller rotational effort, we make uθ converge to zero. Assuming infinitesimal rotations of those axes, uθ can be approximately represented as

(24) $$u\theta =\left(n\times n_{Z}\right)\theta_{n,n_{Z}}+\left\{\begin{array}{cc} \left({\tilde{n}}_{l}\times n_{X}\right)\theta_{{\tilde{n}}_{l},n_{X}} & \theta_{{\tilde{n}}_{l},n_{X}}<\theta_{{\tilde{n}}_{s},n_{X}}\\ \left({\tilde{n}}_{s}\times n_{X}\right)\theta_{{\tilde{n}}_{s},n_{X}} & \mathrm{otherwise} \end{array}\right.$$

(25) $$\theta_{n,n_{Z}}=\arccos\left(\frac{n\cdot n_{Z}}{∥n∥∥n_{Z}∥}\right)$$

(26) $$\theta_{{\tilde{n}}_{l},n_{X}}=\arccos\left(\frac{{\tilde{n}}_{l}\cdot n_{X}}{∥{\tilde{n}}_{l}∥∥n_{X}∥}\right)$$

(27) $$\theta_{{\tilde{n}}_{s},n_{X}}=\arccos\left(\frac{{\tilde{n}}_{s}\cdot n_{X}}{∥{\tilde{n}}_{s}∥∥n_{X}∥}\right),$$

where $n_{X}$ and $n_{Z}$ are the normals of camera axes *X* ([1,0,0]) and *Z* ([0,0,1]), respectively. The first term in Equation (24) aligns camera axis *Z* with the surface normal and the second term aligns camera axis *X* with the long or short axis having the smaller angle respective to axis *X*.

Thus, the gripper-reference translational and rotational velocities can be calculated based on the depth image and a camera-intrinsic parameter matrix. In Equation (23), $d^{*}$ is set to 0.15 m, indicating that the gripper is sufficiently close to the graspable surface for grasping. ρ can be calculated based on Equation (22), where, given the camera-intrinsic parameter matrix, *Z* can be derived from the depth image at a certain point.

The joint reference angular velocities $q_{r}$ of the robot can thus be simply calculated as

(28) $$q_{r}=\int {\dot{q}}_{r}\mathrm{dt}=\int J_{c}^{-1}\left[\begin{array}{c} V\\ \Omega \end{array}\right]\mathrm{dt},$$

where $J_{c}^{-1}$ is the pseudoinverse of the Jacobian matrix in camera coordinates.

## 6. Experiment

To train the DCNN model, we generated 15,000 annotated depth images of a pile of boxes in Gazebo using the framework proposed in Section 4.3. We then conducted experiments of DCNN training based on the artificial dataset. Experiments were conducted on a computer with an Intel Core i7-6700K CPU operating at 4.00 GHz and an Nvidia Geforce GTX 1080Ti GPU. The DCNN was implemented in Keras. The Adam optimizer was used with its suggested default parameters (α=0.0001, $\beta_{1}=0.9$, $\beta_{2}=0.999$). The batch size was set to 16. The precision was calculated to evaluate the DCNN performance.

To validate grasp planning performance, we compared performance for grasp point position predictions with Dex-Net 4.0, which uses only depth images as input. We evaluated performance according to percentage of predicted optimal grasp points with a nearest distance to ground truth surface center points of less than 30 pixels.

**Figure 8:**
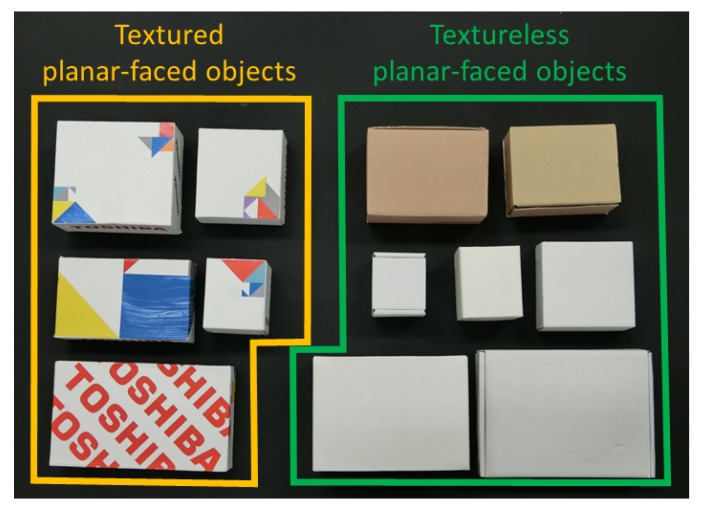
The object set. The yellow region indicates textured boxes. The green region indicates textureless boxes.

To validate efficiency of the proposed robot bin-picking system, experiments involved pick-and-place tasks in which robots picked disorganized planar-faced objects in a bin and placed them on a conveyor, as in Figure 1. The target planar-faced objects were a mix of textureless and textured boxes. As Figure 8 shows, boxes with varying sizes were used in the experiment, including 5 textured and 7 textureless boxes.

Twelve boxes were picked in each of 10 trials. Only a depth image captured by an Intel SR300 camera was used. In each trial, the robot performed grasp attempts until all boxes are removed from the bin. The task was considered complete when no objects remained in the bin, or as failure after 5 unsuccessful grasp attempts for a target box or when the grasp planning loop failed to predict a grasp point at the robot’s home position. The grasp planning loop ran on a computer with an Intel Core i7-4790 CPU operating at 3.60 GHz and an Nvidia GeForce 1080 GPU, while the VS loop ran on a computer with an Intel Core i7-9700K CPU operating at 3.60 GHz. These computers communicated using an ROS (an open-source robotic operating system) communication protocol [45]. The average success rate of ten trials and takt time (pieces per hour (PPH) with successful grasps) were calculated and compared with previous studies.

## 7. Results

Table 1 shows the precision of DCNN model prediction. Precision for the top 1 and top 1% predictions exceed 80%, which can be considered as a reliable level of output.

Table 2 shows percentages of grasp points with distance to a surface center of less than 30 pixels. Both Dex-Net and our grasp planning method can predict surface grasp points, but our method is more likely to predict ones close to the surface center (79.16% for our method vs. 58.88% for Dex-Net).

**Figure 9:**
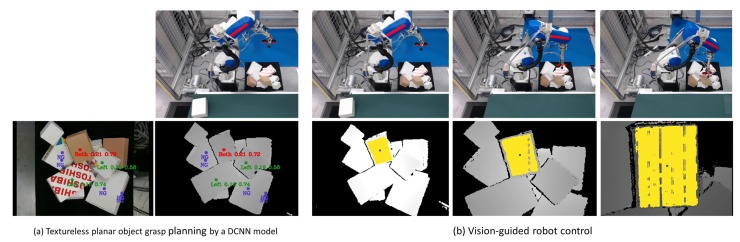
Example of one grasp attempt. (**a**) Grasp planning for textureless planar-faced objects by a DCNN model. Note that RGB color is only for visualization of the results. Text in grasp point labels indicates the grasp pattern class. First and second values beside the text are $J_{\mathrm{distance}}$ and $J_{\mathrm{inclination}}$, respectively. (**b**) Vision-guided robot control. Yellow areas are target surfaces and blue points are surface centers.

Further, our system achieved a 97.5% success rate at a speed exceeding 1000 PPH, as demonstrated in the video (Supplementary File 1) in Supplementary Material. Both textured and textureless boxes could be held by a suction hand. Figure 9 shows examples of successful grasp attempts. The grasp planning loop successfully output the best grasp point with its position and grasp pattern at the robot home position. The VS loop then started to track and access the surface containing the point. Although the DCNN model was trained on a synthesized depth image, refinement of the DCNN prediction by the surface descriptor makes the picking system efficient. Table 3 compares average success rates and takt times with those of previous studies.

## 8. Discussion

This study aimed to propose a novel depth image–based vision-guided robotic bin-picking system for textureless planar-faced objects utilizing a two-vacuum-cup suction hand. To the best of our knowledge, no previous studies have proposed a DCNN model for predicting grasp patterns (vacuum cup activation modes) for a two-vacuum-cup suction hand.

Experimental results demonstrated that the proposed bin-picking system can efficiently complete pick-and-place tasks for textureless planar-faced objects with a 97.5% success rate at speeds exceeding 1000 PPH. The DCNN model can predict the grasp point position and its corresponding proper grasp pattern. A surface feature descriptor further refines DCNN output and calculates surface features to make the VS free from texture features.

Only three box types were used during DCNN model training, but the robot could pick all 12 box types in the experiment, indicating that a DCNN model might be able to predict grasp points for novel boxes because they have similar convolutional features.

### 8.1. Comparison with Previously Proposed Bin-Picking Systems

The proposed system has been compared with previously proposed bin-picking systems [33,46,47,48] by grasp planning performance and overall system efficiency.

#### 8.1.1. Grasp Planning Performance

**Figure 10:**
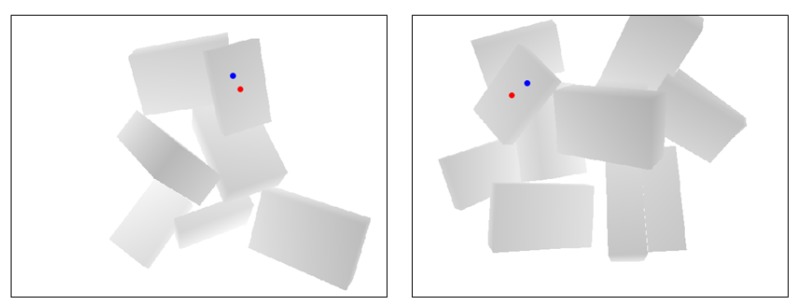
Planned optimal grasp point. The red and blue points are our grasp planning method and Dex-Net 4.0 outputs, respectively.

We compared grasp planning performance using the proposed method with that using Dex-Net 4.0 [46], because both systems use only depth images. Both output grasp point positions (though Dex-Net does not output grasp patterns), so we evaluated performance according to the percentage of predicted optimal grasp points with a nearest distance to a ground-truth surface center point of less than 30 pixels. The results indicate that our method is more likely to predict a grasp point close to the surface center (see output examples of the two systems in Figure 10). These results agree with previous results [47] showing that Dex-Net may perform poorly at predicting grasp points near the center of mass of an object. Note that we did not perform a comparison with [47,48] because color images are indispensable for the grasp planning method described there.

#### 8.1.2. Overall System Efficiency

We compared the efficiency of our system with data reported in [33,46,47,48]. Our system outperforms previously proposed bin-picking systems [33,46,47,48] in terms of grasp planning processing times and mean PPH. This comparison is reasonable despite the different object sets for two reasons. First, the time cost of grasp planning depends on only the input image size and CNN model complexity, so it will not change with object shape complexity. Also, those systems would likely have improved success rates if they were applied to box-picking tasks, because their object sets include objects with complicated shapes and success rates may increase when picking objects with simple shapes. However, we assume that success rate levels of 95% or higher are acceptable for bin-picking of common objects (e.g., boxes) in a warehouse. The main advantage of our system is that it can achieve high success rates at high speed (1000 PPH, which is approximately three times faster than Dex-Net) for picking planar-faced objects with rectangular surface contours.

Compared with Dex-Net [33,46], our system achieves the same level of success rates at higher speeds. Dex-Net 4.0 trains a GQ-CNN to predict grasp probabilities of grasp candidates for a one-cup-suction hand and a two-finger-gripper. Then, the highest-quality grasp candidate and its corresponding robotic arm are selected to grasp the object. In such multifunction robotic systems and picking methods (grasping or suction), selection policy learning allows manipulation of objects with complex shapes. However, Dex-Net 3.0 and Dex-Net 4.0 use a cross-entropy method to iteratively search over the policy action space for the best grasp, which may result in higher computation costs than that of our one-shot grasp detection method.

Zeng et al. [47] also used a multifunction robotic hand, defining four motion primitives and training a multimodal CNN to predict grasp affordances for each. However, their system requires both RGB and depth images and preparation of grasp affordance heatmaps for predefined motion primitives, which might be difficult in a warehouse. We suggest that when focusing on textureless planar-faced objects in warehouse picking tasks, learning the grasp points of graspable surfaces rather than pixel-wise learning of an affordable map is sufficient. Training dataset preparation will therefore become easier because pixel-wise labeling is unnecessary.

Another recent study [48] proposed a planar-faced object bin-picking system by a two-stage grasp planning method, conducting experiments on USB flash drive packs as representative thin planar objects. They first used a CNN to segment the packs and classify their front and back sides, then estimated object poses to plan a grasp. They achieved a very high success rate (99.6%), but required a longer processing time (0.9 s) than ours. The probable reason for higher processing speeds in our system is that it directly detects grasp points without segmentation. Further, that study only dealt with one type of planar object (the same USB flash drive pack), while our system can grasp planar-faced objects with varying size.

Further, those systems [33,46,47,48] used one-cup suction hands, while our system uses a two-vacuum-cup suction hand and learned appropriate activation policies. For objects with larger planar surfaces in particular, suction by two cups is obviously more stable than that by only one cup.

### 8.2. Benefits of Bin-Picking Policies Considering Grasp Pattern Prediction

An efficient learning-based bin-picking system aims at finding a policy that maximizes PPH. Mahler et al. [46] regarded this as a sequential learning problem in which grasp actions affect future states of the heap, and suggested that the performance of a policy trained by supervised learning is sufficient for sequential picking tasks, as compared with imitation learning and reinforcement learning. Our results agree with their idea, in that a CNN for grasp point position and grasp pattern prediction ranking along with a grasp point ranking process can achieve high efficiency.

The conventional bin picking policy [46] considers collision situations and force analytics. Our method does not consider analytic force information based on the assumption that surface area larger than that of the vacuum cup are considered as graspable. In addition to the collision situation, our predicted grasp pattern also considers robot rotation efforts and camera-center position height. Incorporating these factors improves the efficiency of suction by two vacuum cups. For example, lower rotation efforts with respect to the robot pose at its home position helps the robot access the surface more quickly. Considering camera center height helps the robot to select a grasp pattern with lower collision risk.

### 8.3. Benefits of Depth-Image-Based Visual-Guided Bin-Picking System

One benefit of a depth-image-based visually guided bin-picking system is that it does not require texture features, which are sensitive to environmental brightness; objects imaged under poor lighting conditions may reduce object tracking capabilities by template matching [49] or object recognition by a CNN [50]. Depth features are more robust, because they can be measured by an infrared camera in dark environments.

Another benefit is that depth images are easier to synthesize than color images. For synthesized color images, sim-to-real technologies such as domain randomization are required to improve performance [51]. Our results demonstrated that a DCNN model trained on synthesized depth images can be directly applied to box-bin picking with high performance, given a properly designed descriptor for DCNN model output refinement.

### 8.4. Grasp Failure Analysis

**Figure 11:**
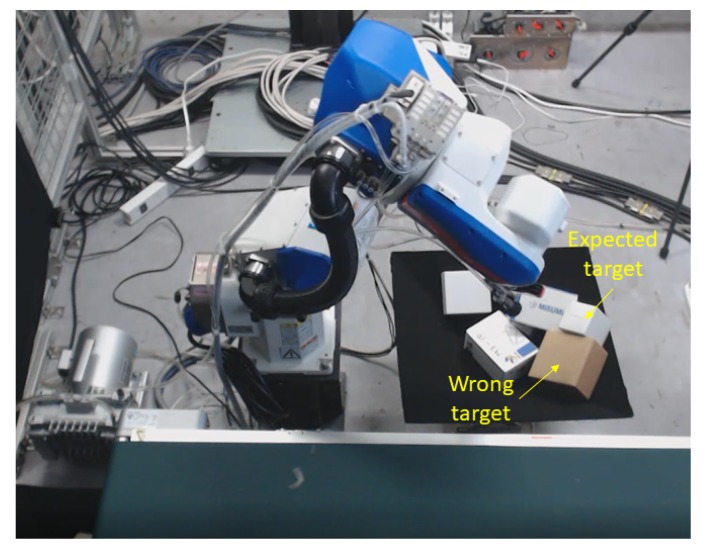
Grasp failure example.

Grasp attempts failed in cases where the targeted box was adjacent to another box as shown in Figure 11, where the expected target object is the upper box but the robot targets the wrong box because its surface inclination angle is smaller than that of the expected target. Our policy (Equation 7) prioritizes surface inclinations with angles exceeding 30 degrees and ignores the evaluation of surface height. One method to resolve this problem would be to additionally evaluate differences between surface inclination angles, conducting further height evaluations if the difference is smaller than some threshold. We will thus modify this policy in the future work. Failures also occurred when surface areas were close to that of the vacuum cup, possibly causing the cup to only partly contact the surface and thus lowering the suction force to below expectations. One solution to this problem would be to add a safety constraint on the surface area, stipulating that, for example, the target surface area must be at least 1.2 times larger than the cup area.

### 8.5. Limitations and Future Work

This study has several limitations to address in future work. Our picking system is designed for only random bin-picking tasks in which the system cannot grasp a specific object, because we directly detect grasp points without object segmentation and detection. Object segmentation might be required for certain tasks such as picking a specific item ordered by a customer.

In addition, the current implementation can deal with only planar-faced objects with rectangular contours, because the training dataset was generated using only boxes where the lengths of long and short surface sides are required for class labeling (Figure 6). We will improve the class-labeling framework to allow for other objects with planar surfaces but nonrectangular contours, such as circles or polygons, by calculating a bounding box for surface contours and utilizing its size information. Moreover, we will generate the training dataset in a simulator by using various planar-faced objects, including ones with complicated shapes rather than only boxes. However, increased shape complexity may decrease DCNN prediction accuracy, so to address this issue we will utilize more complicated backbones such as ResNet-101 and expand the dataset volume.

Furthermore, sensory noise (e.g., camera depth image noise) and environmental uncertainty may affect accuracy of the DCNN prediction results and the robustness of the visual servoing control scheme. In this study, we used only a post-processing-filter provided by the Intel RealSense SDK, and the experimental results (97.5% grasp success rate) suggest that such image preprocessing is effective. Employing an information fusion process would be one effective way of further improving the system robustness. Recent studies have shown that Kalman filters and particle filters greatly contribute to human hand tracking by compensating for tracking error and sensor noise [52,53,54]. The current picking system uses only one robotic-hand-mounted camera. Using extra fixed cameras and integrating multicamera sensory information via a Kalman filter may improve the system robustness to noise and occlusion. Another possibility is to adopt a sliding mode control to improve object tracking robustness while considering unknown disturbances and uncertainties [55].

Another limitation is that experimental conditions such as the relative locations of robots and bins may affect bin-picking task efficiency. Future work will therefore investigate the efficiency of the system under different experimental conditions.

## Figures and Tables

**Table 1:** Precision of DCNN model prediction.

Top 1	Top 1%	Top 5%	Top 10%
83.10%	80.25%	67.22%	55.64%

**Table 2:** Percentage of grasp points with a distance to a surface center of less than 30 pixels.

Dex-Net 4.0	Ours
58.88%	79.16%

**Table 3:** Comparison of bin-picking system performance.

System	Object Set	Robotic Hand	Input	Success Rate (%)	grasp Planning Time Cost (s)	Mean Piece per Hour (PPH)
Dex-Net 3.0 [33]	Prismatic solids	Suction (1 cup)	Depth	98	About 3.0	-
Dex-Net 4.0 [46]	Level 1 objects	Suction (1 cup) + gripper (2 fingers)	Depth	97 *	-	Over 300
Zeng et al. [47]	APC dataset	Suction (1 cup) + gripper (2 fingers)	RGB + depth	58.3	0.06	-
Le et al. [48]	USB flash drive packs	Suction (1 cup)	RGB + point cloud	**99.6**	0.862	-
Ours	Boxes	Suction (2 cups)	Depth	97.5	**0.034**	**Over 1000**

Level 1 objects consist of prismatic and circular solids (e.g., boxes, cylinders) common in groceries, toys, and medicine. The ARC dataset indicates the Amazon Picking Challenge (ARC) dataset. “*” indicates the success rate and time cost of grasping by a gripper/suction cup hybrid.

## Supplementary Materials

The following are available at https://www.mdpi.com/1424-8220/20/3/701/s1.
